# Efficacy of Low-Dose Fenfluramine on Adult Patients With Lennox-Gastaut Syndrome

**DOI:** 10.7759/cureus.87250

**Published:** 2025-07-03

**Authors:** Mutsuki Takeda, Yu Kitazawa, Masaki Sonoda, Keisuke Morihara, Yosuke Miyaji, Yuichi Higashiyama, Katsuo Kimura, Naohisa Ueda, Hiroshi Doi, Fumiaki Tanaka

**Affiliations:** 1 Department of Neurology and Stroke Medicine, Yokohama City University Graduate School of Medicine, Yokohama, JPN; 2 Department of Neurosurgery, Yokohama City University Graduate School of Medicine, Yokohama, JPN; 3 Department of Neurology, Yokohama City University Medical Center, Yokohama, JPN

**Keywords:** adult, fenfluramine, lennox-gastaut syndrome, low-dose, sigma-1

## Abstract

Purpose

Fenfluramine has received approval for treating seizures in individuals with Lennox-Gastaut syndrome (LGS). Nonetheless, Phase III trials mainly focused on children, leaving limited information regarding its effectiveness and safety in adults. This case series aimed to elucidate these factors in adult patients.

Methods

This case series examined the clinical progression of adult patients diagnosed with LGS who received fenfluramine at our clinic following its approval in Japan in March 2024, extending through February 2025. Fenfluramine was administered to those experiencing drop attacks and generalized slow spike-and-wave complexes, fulfilling the conventional diagnostic criteria for LGS, and their responses to treatment were analyzed.

Results

This case series involved nine adult patients aged 18-49. All patients demonstrated efficacy, with seven (78%) experiencing at least a 50% reduction in seizure frequency. No one needed maintenance doses above the recommended starting minimum of 0.2 mg/kg/day, and five patients (56%) achieved seizure control with even lower doses. Remarkably, three patients who did not meet the latest 2022 diagnostic criteria also demonstrated positive responses. Adverse events were reported in five patients (56%), primarily including somnolence, diarrhea, and anorexia. In these situations, a dose reduction to half was necessary, yet efficacy was maintained.

Conclusion

Low-dose fenfluramine may effectively manage seizures in adult patients with LGS while reducing adverse effects. This case series provides a growing body of real-world data regarding the use of fenfluramine in this patient population.

## Introduction

Lennox-Gastaut syndrome (LGS) is a severe form of epileptic encephalopathy characterized by various drug-resistant seizure types, including tonic, atonic, and atypical absence seizures, along with unique interictal electroencephalographic (EEG) patterns and cognitive and developmental disabilities [1]. The syndrome typically begins between 18 months and 8 years of age, with a peak occurrence between 3 and 5 years. However, onset in the second decade is rare [2]. Drop attacks, typically stemming from tonic, atonic, or clonic seizures, affect more than half of patients. These dangerous incidents can lead to sudden head flexion or falls, frequently resulting in head injuries [3-5].

Lennox-Gastaut syndrome (LGS) is typically characterized by multiple seizure types, with specific EEG patterns such as slow spike-wave (SSW) and generalized paroxysmal fast activity (GPFA), or a combination of both [6]. The International League Against Epilepsy (ILAE) notes that numerous clinicians refer to "LGS” as a general term for severe, early-onset, drug-resistant epilepsies, potentially encompassing other epileptic syndromes within this classification [1].

To address this issue and better differentiate LGS from other early-onset epilepsies with more favorable outcomes, such as epilepsy with myoclonic-atonic seizures, the ILAE released revised diagnostic criteria in 2022 [1]. These criteria define LGS through the following characteristics: (1) several types of drug-resistant seizures commencing before 18 years, with at least one being a tonic seizure; (2) cognitive and often behavioral impairments, which might not emerge at the onset of seizures; and (3) widespread SSW and GPFA on EEG [1]. In contrast to the prior definition, the new criteria are more rigorous, necessitating the occurrence of tonic seizures and verification of both SSW and GPFA on EEG. These criteria emphasize the significance of particular clinical and EEG characteristics, rather than treating “LGS” as a general term for severe epilepsy, to guarantee precise diagnosis, suitable monitoring of disease progression, and access to targeted therapies [1].

A prior study indicated that only 32% of pediatric patients diagnosed with LGS using traditional criteria fully fulfilled the revised ILAE diagnostic criteria [7]. In cases of late-onset LGS, SSW discharges occur less frequently, making it more unlikely for these instances to meet the updated criteria [8]. These results could complicate the application of syndrome-specific treatments, such as rufinamide (RUF) or fenfluramine (FFA), which are typically recommended based on strict syndromic definitions.

FFA was initially created as an appetite suppressant and was extensively used for treating obesity [9]. Its antiseizure properties were first observed in the 1980s [10]. Unlike conventional antiseizure medications (ASMs), FFA exerts its therapeutic effect by enhancing serotonin receptor activity [11,12] and modulating sigma-1 receptors (S1Rs) [13-15]. Nevertheless, it was removed from the market in 1997 due to its association with cardiac valvulopathy and pulmonary hypertension, especially when taken alongside phentermine [16,17]. In the 2010s, small open-label studies demonstrated a marked reduction in seizure frequency in patients with Dravet syndrome (DS) treated with low-dose FFA [18]. These results prompted extensive randomized controlled trials that validated the drug’s effectiveness and safety at much lower doses than those previously used for weight management [19]. In LGS, a randomized, double-blind, placebo-controlled Phase III trial demonstrated that FFA significantly reduced the frequency of drop seizures compared to placebo in patients with treatment-resistant LGS (median age: 13 years; range: 2-35), with an acceptable safety and tolerability profile [20].

In Japan, FFA received approval in March 2024 as an antiseizure medication (ASM) for treating LGS, after its prior approval for DS. The recommended initial dose is 0.2 mg/kg/day, with a maximum dose of 0.7 mg/kg/day. An open-label extension study reinforced the sustained efficacy, including a significant reduction in drop seizures, and safety of FFA within this dosage range among patients with LGS, including adults [21]. Clinical trials indicate that the therapeutic effects seen in adults tend to be less pronounced than those in pediatric populations. However, there is a lack of real-world data on the application of FFA in adult patients with LGS. This case series aims to offer practical, real-world clinical insights into the use of FFA in adult patients with LGS.

## Materials and methods

Demographic data for this case series were obtained from Yokohama City University Hospital in Japan. The criteria for inclusion were: (1) patients aged 18 or older diagnosed with LGS; (2) the start of FFA treatment between March 2024 and February 2025, right after its approval in Japan; and (3) an observation period of at least 2 months. At our clinic, LGS diagnoses were based on traditional criteria, which include multiple seizure types, cognitive impairment, and EEG findings including SSW.

Clinical data were extracted retrospectively from medical records spanning March 2024 to May 2025. The information gathered comprised age, sex, age at seizure onset, underlying causes, cognitive function, EEG results, seizure frequency and types, medication history, FFA dosage, side effects, and rates of seizure reduction. To address missing data, supplementary information was gathered through in-person or phone interviews with caregivers during routine follow-up visits. Informed consent was secured from all adult participants or their legal guardians. This case series received approval from the Institutional Review Board of Yokohama City University.

## Results

Clinical features and results

Following its approval in Japan, FFA was given to nine adult patients (three females and six males) with LGS from March 2024 to February 2025 (Table 1). The median age at the start of FFA treatment was 26 years (18-49 years). All patients experienced various seizure types, including drop attacks, tonic seizures, myoclonic seizures (MS), and generalized tonic-clonic seizures (GTCS). EEG results confirmed the presence of SSW and/or GPFA in all patients.

**Table 1:** Patient profile. F: female; M: male; Y: yes; N: no; SSW: slow spike-and-wave complexes; GPFA: generalized paroxysmal fast activity; ASM: antiseizure medication; TSC: tubular sclerosis complex; HIE: hypoxic-ischemic encephalopathy; AZM: Acetazolamide; CBZ: carbamazepine; CLB: clobazam; CZP: clonazepam; LCM: lacosamide; LTG: lamotrigine; LEV: levetiracetam; PB: phenobarbital; PER: perampanel; RUF: rufinamide; TPM: topiramate; VPA: valproic acid; ZNS: zonisamide.

Case	1	2	3	4	5	6	7	8	9
Age (years)	18	21	18	18	34	26	49	22	37
Sex	M	M	F	M	M	M	F	M	F
Onset (years)	3	0	1	0	14	3	11	1	0
Etiology	Mycoplasma pneumoniae encephalitis	West syndrome; TSC	22q13.3 deletion	West syndrome; TSC	Unknown	Unknown	Unknown	HIE	West syndrome; TSC
Mental retardation	Y	Y	Y	Y	Y	Y	Y	Y	Y
SSW	Y	Y	Y	Y	Y	Y	Y	Y	Y
GPFA	N	Y	Y	Y	Y	Y	Y	Y	N
Current seizure types (frequency)									
Drop attack	Y (daily)	Y (daily)	Y (daily)	Y (daily)	Y (monthly)	Y (daily)	Y (daily)	Y (daily)	Y (weekly)
Tonic	Y (daily)	Y (daily)	N	Y (daily)	Y (monthly)	Y (daily)	Y (daily)	Y (daily)	Y (weekly)
Generalized tonic clonic	N	Y (daily)	N	Y (monthly)	N	Y (daily)	N	N	Y (monthly)
Others	Myoclonic seizure: daily	Myoclonic seizure: daily	Atypical absence: daily	N	Atypical absence: monthly	N	Impaired awareness: daily	Impaired awareness: unknown	N
Existing ASMs	CLB; LTG; TPM; VPA	CLB; LCM; LTG; VPA	CBZ; CLB; RUF; VPA	CZP; LTG; VPA; ZNS	CLB; LCM; LTG; LEV; TPM; VPA	CLB; LTG; RUF; VPA	CBZ; CLB; CZP; LEV	PB; PER; VPA; ZNS	AZM; CLB; CZP; LEV; VPA; ZNS
Other treatments	-	Everolimus	Corpus callosotomy	-	-	-	-	-	Everolimus

Three patients (33%) were diagnosed with tuberous sclerosis complex (TSC), each with a previous history of West syndrome that later progressed to LGS. One patient presented with a 22q13.3 deletion. All patients exhibited moderate to severe cognitive impairment or intellectual disability.

All patients were treated with several ASMs before starting FFA, averaging five medications simultaneously. Frequently prescribed ASMs included valproic acid (VPA), clobazam (CLB), lamotrigine (LTG), topiramate (TPM), and RUF. One patient had a corpus callosotomy, and two were treated with everolimus for TSC. Notably, everolimus demonstrated effectiveness in managing status epilepticus in a specific case (Case 9).

FFA was started at doses between 0.05 and 0.2 mg/kg/day, with adjustments made based on clinical response and tolerability (Table 2). All patients experienced a decrease in seizure frequency. Seven patients (78%) achieved a reduction of more than 50% in overall seizure frequency. GTCS displayed the most significant response, with reductions ranging from 80% to 100% in Cases 2, 4, 6, and 9. Remarkably, seizure frequency diminished across all types: drop attacks decreased by 25%-100 % for all patients, MS by 25%-50 % (Cases 1 and 2), and atypical absences by 80% (Case 5). None of the patients required maintenance doses above the recommended minimal starting dose of 0.2 mg/kg/day, and seizure control was achieved at even lower doses (0.07-0.1 mg/kg/day) in five patients (56%). A follow-up EEG has not been performed.

**Table 2:** Clinical response and tolerability of fenfluramine. *: The follow-up period in Case 3 includes a three-month of temporary suspension of fenfluramine.

Case	1	2	3	4	5	6	7	8	9
Fenfluramine									
Initial dose (mg/kg/day)	0.2	0.1	0.2	0.1	0.1	0.05	0.05	0.05	0.1
Maintenance dose (mg/kg/day)	0.1	Discontinued	0.2	0.07	0.2	0.1	0.2	0.1	0.1
Follow up period (months)	9	8	11^*^	8	8	4	3	3	2
Adverse effect	Somnolence; body leaning	Diarrhea; anorexia; restlessness	Development of a new seizure type	Somnolence; anorexia; weight loss	None	Diarrhea	None	None	None
Seizure reduction									
Drop attack including tonic	50%	50%	80%	25%	100%	50%	25%	25%	80%
Generalized tonic clonic	n/a	80%	n/a	100%	n/a	80%	n/a	n/a	80%
Others	Myoclonic seizure: 50%	Myoclonic seizure: 25%	Atypical absence: 25%	n/a	Atypical absence: 80%	n/a	Impaired awareness: 50%	Impaired awareness: unknown	n/a

Adverse effects occurred in five patients (56%), with the most prevalent being somnolence (Cases 1 and 4), anorexia (Cases 2 and 4), and diarrhea (Cases 2 and 6). Generally, these effects were dose-dependent and reversible upon modification of the dose. However, in Case 2, worsening anorexia and reduced oral intake necessitated the discontinuation of FFA. In Case 3, treatment was temporarily halted due to the onset of a new type of seizure. FFA was eventually reintroduced, resulting in a decrease in seizure frequency. No cardiac-related adverse effects were noted during the observation period. This case series presents the detailed clinical findings and treatment courses of the following cases.

Case 1

An 18-year-old male with a history of GTCS since he was three initially received treatment with VPA. At the age of eight, he suffered acute *Mycoplasma pneumoniae* encephalitis, which caused a relapse of GTCS, right-sided hemiplegia, and cognitive deficits. By nine, he started experiencing daily MS and drop attacks, which continued despite starting CLB at 13 years, and LTG and TPM at 16 years. EEG results displayed SSW complexes, indicative of LGS. At 18, FFA treatment began at a dosage of 0.2 mg/kg/day, leading to a rapid 50% decrease in seizure frequency. However, side effects such as somnolence and body leaning necessitated a temporary halt in FFA, after which his seizure recurred. Upon resuming FFA at 0.05 mg/kg/day and later increasing to 0.1 mg/kg/day, the patient saw another 50% reduction in MS and drop attacks over nine months.

Case 2

A 21-year-old man had a history of epileptic spasms beginning at four months old and was diagnosed with West syndrome related to TSC at eight months. At age 15, he experienced a marked increase in GTCS. He developed drop attacks and MS. Despite receiving treatment with CLB, lacosamide (LCM), LTG, VPA, and everolimus, he continued to suffer from daily drop attacks and MS. EEG results showed SSW patterns and GPFA, indicating LGS. At age 21, he began treatment with FFA at a dose of 0.1 mg/kg/day, leading to a 50% reduction in seizure frequency within a month. Although diarrhea occurred as a side effect, it resolved after the dose was reduced to 0.05 mg/kg/day. The dose was later increased back to 0.1 mg/kg/day, maintaining reduced seizure frequency for eight months (50% reduction in drop attacks, 80% in GTCS, and 25% in MS). FFA was titrated up to 0.3 mg/kg/day during treatment, but this worsened symptoms of anorexia and restlessness. Following a decrease in dosage to 0.1 mg/kg/day, FFA was ultimately discontinued as the patient could not maintain adequate oral intake.

Case 3

**Figure 1:**
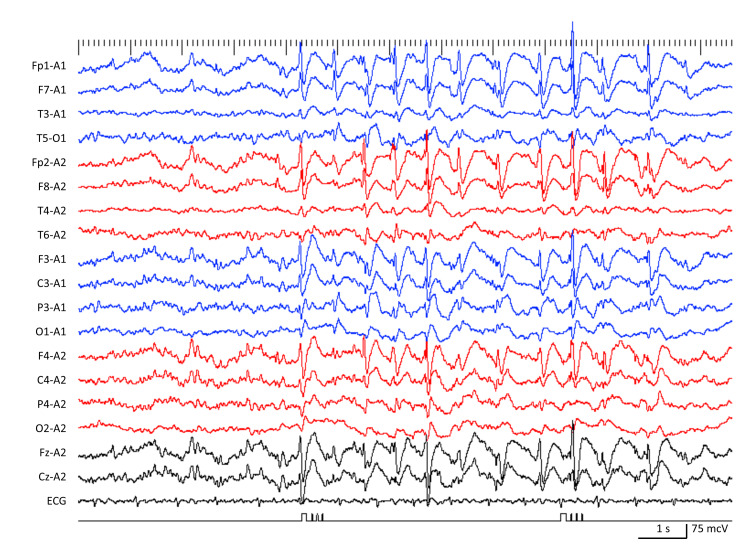
EEG showing slow spike-and-wave complexes in Case 3.

An 18-year-old woman, with a background of complex febrile convulsions at ages one, two, and four, experienced drop attacks at the age of five and was prescribed carbamazepine (CBZ). Genetic analysis indicated a deletion syndrome at 22q13.3. Her seizures were relatively controlled until she turned 13, when the frequency of drop attacks increased, accompanied by atypical absence seizures. Despite adding VPA, CLB, and rufinamide (RUF), her seizure management remained ineffective, leading to multiple daily drop attacks and atypical absence episodes. Long-term video EEG monitoring (LVEM) uncovered atypical absence seizures, drop attacks from atonic seizures, SSW, and GPFA, confirming a diagnosis of LGS (Figure 1). At 18, FFA was started at 0.2 mg/kg/day but was halted soon after due to fine body tremors appearing as a new seizure type. Three months later, she had a corpus callosotomy, but this did not alter her seizure frequency. Two months post-surgery, FFA was restarted at 0.2 mg/kg/day, leading to an 80% decrease in drop attacks and a 25% reduction in atypical absence seizures over six months.

Case 4

An 18-year-old man with a history of epileptic spasms beginning at three months was diagnosed with West syndrome related to TSC and had epilepsy resistant to VPA, vitamin B6, and ACTH therapies. EEG results showing SSW and GPFA suggested LGS as a possibility. Despite being treated with VPA, LTG, zonisamide (ZNS), and clonazepam (CZP), he still experienced daily tonic and atonic seizures, along with occasional GTCS. At the age of 18, FFA was started at a dosage of 0.1 mg/kg/day, leading to a 50% decrease in seizure frequency within a month and a half. The dose was later reduced to 0.05 mg/kg/day to lower side effects such as somnolence, anorexia, and weight loss. Three months afterward, the dosage was increased to 0.07 mg/kg/day, resulting in a 25% reduction in drop attacks and a complete elimination of GTCS for four months.

Case 5

**Figure 2:**
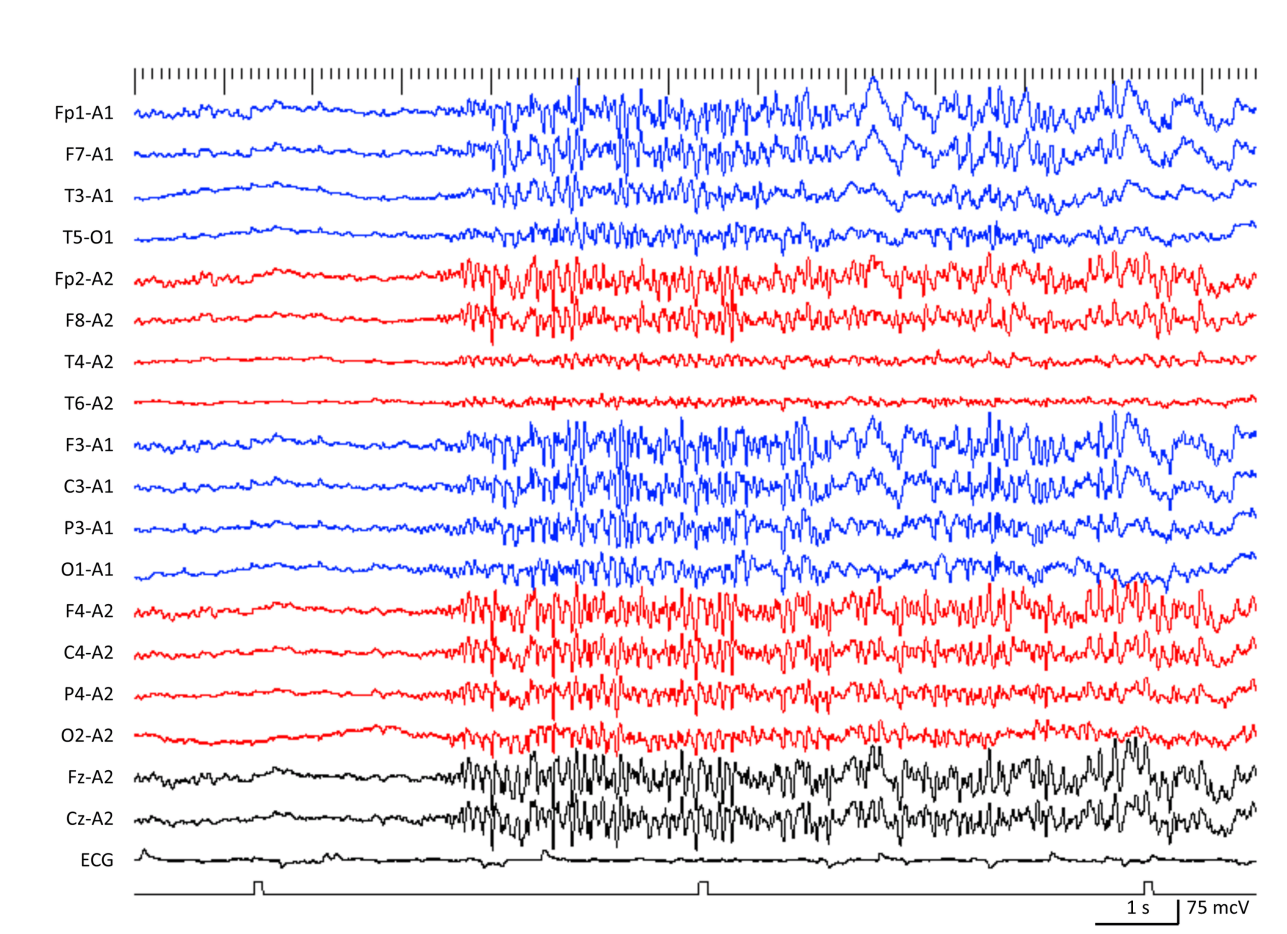
EEG showing generalized paroxysmal fast activity in Case 5.

A 34-year-old man with an intellectual disability began experiencing GTCS at the age of 14. His seizures were effectively managed with VPA and CBZ until he was 27, when the discontinuation of these medications led to atypical absence seizures and drop attacks. He continues to have monthly drop attacks, despite the introduction of LTG, CLB, TPM, LCM, and levetiracetam (LEV). At the age of 34, LVEM indicated the presence of atypical absence seizures, tonic seizure-induced drop attacks, GTCS, SSW patterns, and GPFA, thus confirming a diagnosis of LGS (Figure 2). Subsequently, treatment with FFA was started at a dose of 0.1 mg/kg/day and was increased to 0.2 mg/kg/day after two months. This resulted in a 100% reduction in drop attacks and an 80% decrease in atypical absence seizures, with these improvements occurring without any side effects, and the benefits continued for another six months.

Case 6

A 26-year-old male with normal growth and development started experiencing multiple febrile and afebrile seizures at three years old. He received a diagnosis of epilepsy and was treated with CBZ, CZP, VPA, and intramuscular thyrotropin-releasing hormone. Despite this, he continued to experience daily GTCS and began having daily tonic and atonic seizures as well. After the onset of epilepsy, he also developed an intellectual disability. An EEG showed SSW patterns and GPFA, confirming a diagnosis of LGS. Treatments including ethosuximide, LTG, and RUF were ineffective, although RUF led to a significant reduction in his daytime seizures. At age 26, treatment with felbamate (FBM) began at a starting dose of 0.05 mg/kg, which was gradually increased to 0.1 mg/kg/day. This resulted in approximately a 50% decrease in tonic seizures and an 80% decrease in GTCS over four months. Mild diarrhea was observed as a side effect, but it was not severe enough to necessitate stopping the therapy.

Case 7

A 49-year-old woman began experiencing GTCS, tonic seizures, and impaired awareness seizures at age 11. After starting treatment with CBZ and clobazam (CZP), she remained free from GTCS but still experienced monthly tonic and impaired awareness seizures. At age 35, LEV and CLB were added to her treatment, but there was no significant change in the frequency of her seizures. By age 37, her seizures occurred daily. LVEM at age 43 revealed SSW and GPFA, leading to a diagnosis of LGS. RUF treatment was initiated but had to be stopped due to excessive drowsiness. At age 49, a new therapy with FFA started at a dose of 0.05 mg/kg/day, which was increased to 0.2 mg/kg/day after a month. This change resulted in a 25% reduction in attacks and a 50% reduction in impaired awareness seizures over the next two months, without noticeable side effects.

Case 8

A 22-year-old male with a history of hypoxic encephalopathy, who required neonatal intensive care, was discharged without ASM. By the time he was one year and seven months old, he had experienced GTCS, prompting the initiation of treatment with ZNS. At ages three and four, he suffered from episodes of epilepsy. When he turned 17, he started having daily brief tonic seizures and drop attacks instead of GTCS. There were occasional transient episodes of impaired awareness, but their short duration made it hard to track how often they occurred. While valproate (VPA) and perampanel (PER) were added to his treatment, no improvement was seen. At age 22, an EEG showed SSW and GPFA, leading to a diagnosis of LGS. Consequently, treatment with FBM was started at a dose of 0.05 mg/kg/day. However, no therapeutic effects or adverse reactions were noted for one month, prompting an increase to 0.1 mg/kg/day. This resulted in a 25% reduction in drop attacks over the following two months.

Case 9

A 37-year-old woman with severe intellectual disability initially presented with epileptic spasms at six months and began treatment with CZP. By six years old, her EEG revealed hypsarrhythmia, and a head CT showed intracranial calcifications. She was diagnosed with West syndrome secondary to TSC. Despite treatment with ZNS, acetazolamide, CLB, and LEV, she continued to experience weekly brief tonic seizures, drop attacks, and occasionally GTCS that progressed to status epilepticus. Her EEG showed SSW patterns, indicating a diagnosis of LGS. At age 33, treatment with everolimus was started for TSC, leading to control of status epilepticus. At the age of 37, FFA was added at a dose of 0.05 mg/kg/day, resulting in an 80% reduction in drop attacks and GTCS over two months.

## Discussion

This case series involving nine adult patients with LGS found that FFA treatment resulted in significant decreases in various seizure types, including drop attacks and GTCS, even at lower doses. Most patients noted a reduction in seizure frequency of over 50%.

Nine patients met the traditional ILAE criteria for LGS; however, only six (67%) patients fully met the ILAE 2022 criteria because of the absence of GPFA in Cases 1 and 9 and the absence of tonic seizures in Case 3. This observation aligns with an earlier study that examined potential limitations of the new LGS definition [7]. It suggests that rigid adherence to the updated ILAE criteria for adults may prevent some patients from receiving potentially beneficial FFA treatment. Earlier clinical trials for LGS, including the FFA trial, were based on traditional criteria [7,20].

While adolescent-onset LGS is uncommon, two instances in our study (Cases 5 and 7) were identified as late-onset LGS [2]. In Case 5, seizures commenced at 14, initially diagnosed as epilepsy of unknown cause. However, at age 34, the patient was recently reclassified as having LGS based on LVEM findings, thereby meeting both the ILAE 2022 diagnostic criteria and the traditional diagnostic criteria. Although SSW were noted in our late-onset cases, their overall frequency has been reported as low, and clinical characteristics frequently differ from those in early-onset LGS [8]. As a result, both pediatric and adult patients with late-onset LGS may not fulfill the updated diagnostic criteria, which could restrict their access to syndrome-specific treatments, such as FFA or RUF.

Notably, none of the patients required a maintenance dose higher than the minimum recommended 0.2 mg/kg/day. Five patients (56%) achieved sufficient seizure control at doses below this threshold. The characteristic seizure types and EEG patterns in LGS evolve [22]. GTCS, rather than drop attacks, are most frequently observed in the later stages of LGS [4]. In four patients (Cases 2, 4, 6, and 9) with habitual GTCS, FFA administration resulted in an 80%-100 % reduction in seizure frequency at doses of 0.2 mg/kg or less. In the Phase III study on FFA, the mean age of the patients was 13 years, and GTCS episodes were more responsive to FFA than the other seizure types [20]. Our observations suggest that FFA is highly effective in suppressing GTCS in adults with LGS, even at low doses. Furthermore, drop attacks (in all patients) and MS (in Cases 1 and 2) were reduced with FFA at doses of 0.2 mg/kg or below. However, additional research is necessary to establish the optimal dosing and the efficacy of FFA based on seizure type in adults with LGS.

The primary adverse effects were diarrhea, somnolence, and anorexia. No cardiac events were reported, consistent with the results of an earlier study [21]. Higher doses are more likely to induce adverse effects, suggesting that low-dose maintenance is preferable [23]. However, even at the minimum dose of FFA, one patient (Case 2) experienced marked loss of appetite, leading to treatment discontinuation.

FFA enhances serotonergic neurotransmission by increasing central serotonin release and inhibiting presynaptic serotonin reuptake, thereby increasing serotonin levels in the synaptic cleft. In addition, it positively modulates S1R, a chaperone protein abundant in mitochondria-associated endoplasmic reticulum membranes. This agonistic effect on S1R may help restore the balance between the inhibitory GABA system and excitatory glutamate system, resulting in reduced seizure activity [13,14,20,24,25]. At low doses, S1R agonists bind to the receptor monomers and dimers to activate S1R. However, at higher doses, they bind to S1R higher-order oligomers, which are preferentially bound by antagonists, leading to overall reduced S1R responsiveness [26]. Notably, in the Phase III trial on FFA, there was no significant difference in the proportion of responders at 50% between the 0.2-0.7 mg/kg groups [20]. Furthermore, in a Phase II exploratory clinical trial of pridopidine, an S1R agonist for Huntington’s disease, therapeutic effects were observed at low doses but not at higher doses [27]. Similarly, the maximum impact of igmesine on depression was observed at low doses [28]. Therefore, the minimum recommended dose or low-dose FFA may provide sufficient therapeutic efficacy in adults with LGS. A case report of Dravet syndrome demonstrated the effectiveness of low-dose FFA [29].

About 25% of children diagnosed with severe generalized epilepsy pass away within 20 years, with an average lifespan of 12 years [4]. LGS is associated with a notably high level of treatment resistance, resulting in infrequent successful transitions to adult neurology care [30]. Consequently, adult cases of LGS are uncommon, and the limitation of data collected from a single center further reduces the sample size, posing a significant constraint on this study. Additionally, there may be survivor selection bias: those who reach adulthood could represent a less severe phenotype, likely to explain the favorable seizure control seen with low-dose FFA. Furthermore, as this is a single-center case series with a small sample size, the report lacks the methodological rigor needed to draw definitive conclusions about the efficacy of low-dose FFA in adult LGS. Notably, the observation period ranged from 2-11 months, and some patients had relatively brief follow-up times, which may partially explain the consistent seizure reduction observed. There is also the potential that longer follow-up could uncover seizure recurrence, a need for FFA dose increases, or cessation due to adverse effects.

Although the impact of ASMs on EEG findings has not been consistently demonstrated in previous studies, follow-up EEG data were not available to assess the effectiveness of FFA in our cohort [31]. A recent report suggested that a 10% reduction in GPFA burden correlates with a comparable reduction in seizure frequency, as recorded in seizure diaries [32]. Therefore, longitudinal EEG evaluations should be considered in future studies to better characterize the effects of treatment.

## Conclusions

This case series involving nine patients demonstrated that low-dose FFA effectively controlled seizures in adults with LGS, regardless of whether the onset was early or late, and included individuals who did not fully meet the updated diagnostic criteria for LGS. Our findings offer significant real-world insights into the application of FFA for adult patients with LGS. Nonetheless, to extrapolate these clinical observations to a broader population, multicenter, prospective, long-term studies with larger sample sizes and thorough EEG evaluations are necessary.

## References

[1] Specchio N, Wirrell EC, Scheffer IE (2022). International League Against Epilepsy classification and definition of epilepsy syndromes with onset in childhood: position paper by the ILAE Task Force on Nosology and Definitions. Epilepsia.

[2] Goldsmith IL, Zupanc ML, Buchhalter JR (2000). Long-term seizure outcome in 74 patients with Lennox-Gastaut syndrome: effects of incorporating MRI head imaging in defining the cryptogenic subgroup. Epilepsia.

[3] Asadi-Pooya AA (2018). Lennox-Gastaut syndrome: a comprehensive review. Neurol Sci.

[4] Camfield PR (2011). Definition and natural history of Lennox-Gastaut syndrome. Epilepsia.

[5] Markand ON (2003). Lennox-Gastaut syndrome (childhood epileptic encephalopathy). J Clin Neurophysiol.

[6] (1989). Proposal for revised classification of epilepsies and epileptic syndromes. Commission on Classification and Terminology of the International League Against Epilepsy. Epilepsia.

[7] Asadi-Pooya AA (2023). The new International League Against Epilepsy (ILAE) definition of Lennox-Gastaut syndrome: practical implications and limitations. J Clin Neurosci.

[8] Smith KM, Britton JW, Cascino GD (2018). Late-onset Lennox-Gastaut syndrome: diagnostic evaluation and outcome. Neurol Clin Pract.

[9] Munro JF, Seaton DA, Duncan LJ (1966). Treatment of refractory obesity with fenfluramine. Br Med J.

[10] Aicardi J, Gastaut H (1985). Treatment of self-induced photosensitive epilepsy with fenfluramine. N Engl J Med.

[11] Kannengiesser MH, Hunt PF, Raynaud JP (1976). Comparative action of fenfluramine on the uptake and release of serotonin and dopamine. Eur J Pharmacol.

[12] Baumann MH, Bulling S, Benaderet TS (2014). Evidence for a role of transporter-mediated currents in the depletion of brain serotonin induced by serotonin transporter substrates. Neuropsychopharmacology.

[13] Frampton JE (2023). Fenfluramine: a review in Dravet and Lennox-Gastaut syndromes. Drugs.

[14] Sourbron J, Lagae L (2023). Fenfluramine: a plethora of mechanisms?. Front Pharmacol.

[15] Martin P, de Witte PA, Maurice T, Gammaitoni A, Farfel G, Galer B (2020). Fenfluramine acts as a positive modulator of sigma-1 receptors. Epilepsy Behav.

[16] Rothman RB, Baumann MH, Savage JE, Rauser L, McBride A, Hufeisen SJ, Roth BL (2000). Evidence for possible involvement of 5-HT(2B) receptors in the cardiac valvulopathy associated with fenfluramine and other serotonergic medications. Circulation.

[17] Connolly HM, Crary JL, McGoon MD, Hensrud DD, Edwards BS, Edwards WD, Schaff HV (1997). Valvular heart disease associated with fenfluramine-phentermine. N Engl J Med.

[18] Schoonjans A, Paelinck BP, Marchau F (2017). Low-dose fenfluramine significantly reduces seizure frequency in Dravet syndrome: a prospective study of a new cohort of patients. Eur J Neurol.

[19] Lagae L, Sullivan J, Knupp K (2019). Fenfluramine hydrochloride for the treatment of seizures in Dravet syndrome: a randomised, double-blind, placebo-controlled trial. Lancet.

[20] Knupp KG, Scheffer IE, Ceulemans B (2022). Efficacy and safety of fenfluramine for the treatment of seizures associated with Lennox-Gastaut syndrome: a randomized clinical trial. JAMA Neurol.

[21] Knupp KG, Scheffer IE, Ceulemans B (2023). Fenfluramine provides clinically meaningful reduction in frequency of drop seizures in patients with Lennox-Gastaut syndrome: Interim analysis of an open-label extension study. Epilepsia.

[22] Piña-Garza JE, Chung S, Montouris GD, Radtke RA, Resnick T, Wechsler RT (2016). Challenges in identifying Lennox-Gastaut syndrome in adults: A case series illustrating its changing nature. Epilepsy Behav Case Rep.

[23] Xu Y, Chen D, Liu L (2024). Optimal dose of fenfluramine in adjuvant treatment of drug-resistant epilepsy: evidence from randomized controlled trials. Front Neurol.

[24] Vavers E, Zvejniece L, Maurice T, Dambrova M (2019). Allosteric modulators of sigma-1 receptor: a review. Front Pharmacol.

[25] Muhle H, Kurlemann G, Lehmann I (2024). Fenfluramine in clinical practice: new therapy option for Dravet and Lennox-Gastaut syndromes. Clin Epileptol.

[26] Maurice T (2021). Bi-phasic dose response in the preclinical and clinical developments of sigma-1 receptor ligands for the treatment of neurodegenerative disorders. Expert Opin Drug Discov.

[27] Toyoda K, Uchiyama S, Yamaguchi T (2019). Dual antiplatelet therapy using cilostazol for secondary prevention in patients with high-risk ischaemic stroke in Japan: a multicentre, open-label, randomised controlled trial. Lancet Neurol.

[28] Benjamin G, Guy D (1999). Involvement of sigma receptors in the modulation of the glutamatergic/NMDA neurotransmission in the dopaminergic systems. Eur J Pharmacol.

[29] Iguchi A, Yamaguchi T, Yabe T (2024). Low-dose fenfluramine as an effective treatment option for 'atypical' Dravet syndrome. Epilepsy Behav Rep.

[30] Camfield P, Camfield C (2007). Long-term prognosis for symptomatic (secondarily) generalized epilepsies: a population-based study. Epilepsia.

[31] Nizami FM, Trivedi S, Kalita J (2024). A systematic review of electroencephalographic findings in Lennox-Gastaut syndrome. Epilepsy Res.

[32] Dalic LJ, Warren AE, Spiegel C, Thevathasan W, Roten A, Bulluss KJ, Archer JS (2022). Paroxysmal fast activity is a biomarker of treatment response in deep brain stimulation for Lennox-Gastaut syndrome. Epilepsia.

